# Supplementary material for: Extracellular Vesicles Potentiate Medulloblastoma Metastasis in an EMMPRIN and MMP-2 Dependent Manner
Source: Cancers (Basel). 2023 May 4;15(9):2601. doi: 10.3390/cancers15092601 (PMC10177484; doi:10.3390/cancers15092601)
Supplement: Supplementary file 1 [file cancers-15-02601-s001.zip › cancers-2339075-supplementary_bc.pdf]

Supplementary figures

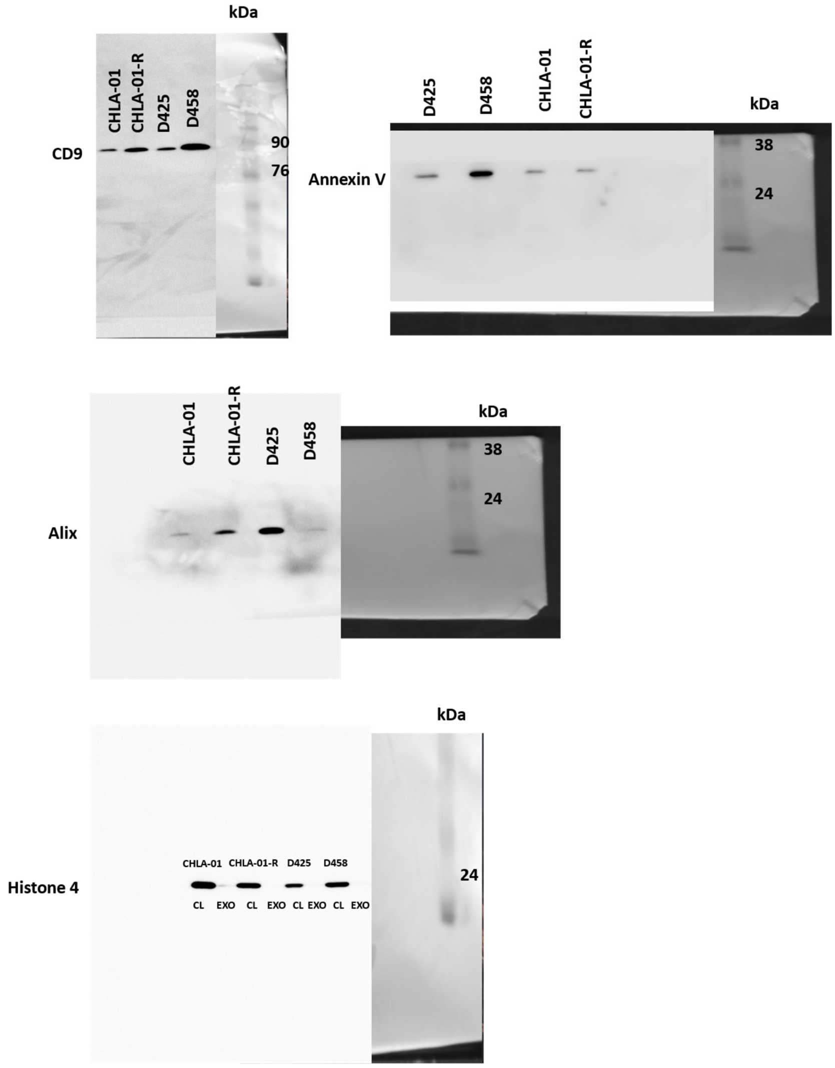

Supplementary Figure S1. Characterisation of exosomes from medulloblastoma cell lines. Representative full length western blot images from Figure 1A and Aii confirming that exosomes express EV markers CD9, Annexin V and Alix and are absent for Histone 4. Exo= exosomes, CL= cell lysates.

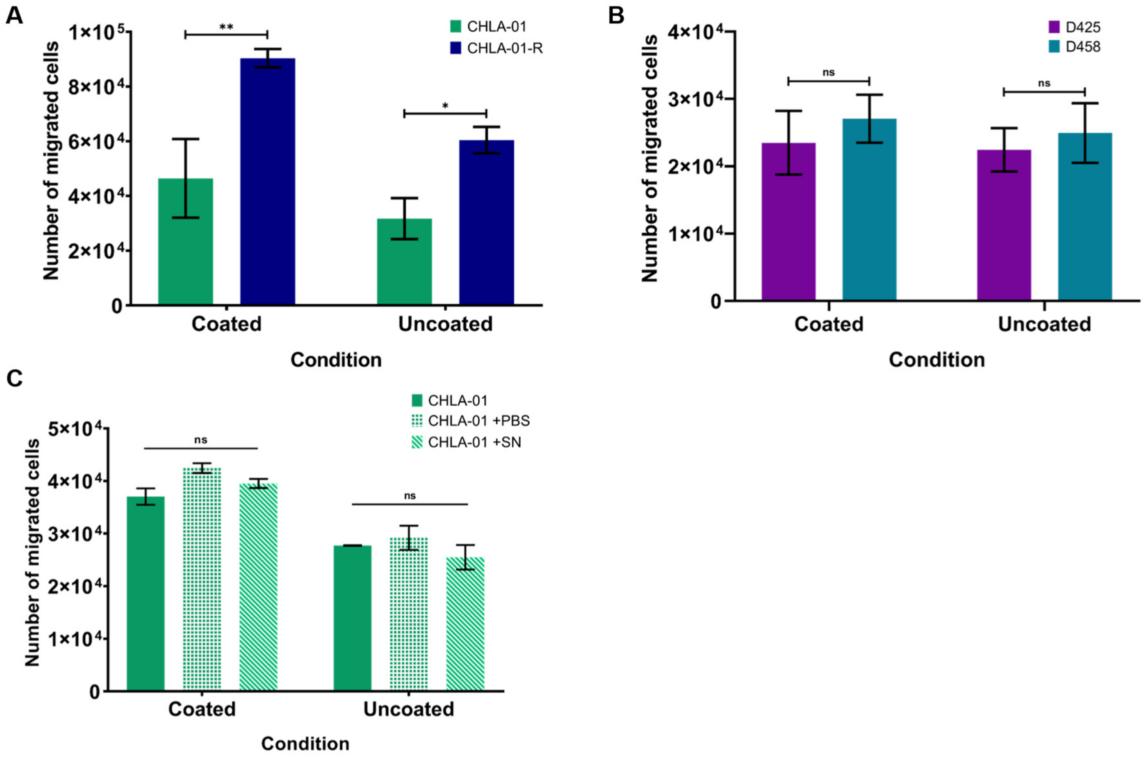

Supplementary Figure S2. Comparisons of cell line migration and invasion in transwell assays. Cell lines were seeded in a coated (to measure invasion) or uncoated (measure migration) transwell assay. After 24 hours cells were collected from the underside of the transwell and from the lower chamber and cell numbers were quantified using a metabolic activity assay. (A) There was a significant increase in cell migration of metastatic CHLA-01R cells compared to primary CHLA-01 cells in both the coated and uncoated transwell assay. (B) In the group 3 cell lines this increase did not achieve significance. (C) Primary CHLA-01 cell lines were co-cultured with media, PBS or vesicle-free supernatant (SN) for 24 hours prior to seeding in the transwell assay. No significant difference in migration between any of the conditions were observed. Results are shown as mean ± standard error of at least three biological replicates. Significance differences in migration was calculated using one-way ANOVA analyses with Dunnett's multiple comparisons post-hoc test, (*$p \leq 0.01$,**$p \leq 0.01$) (ns=not significant).

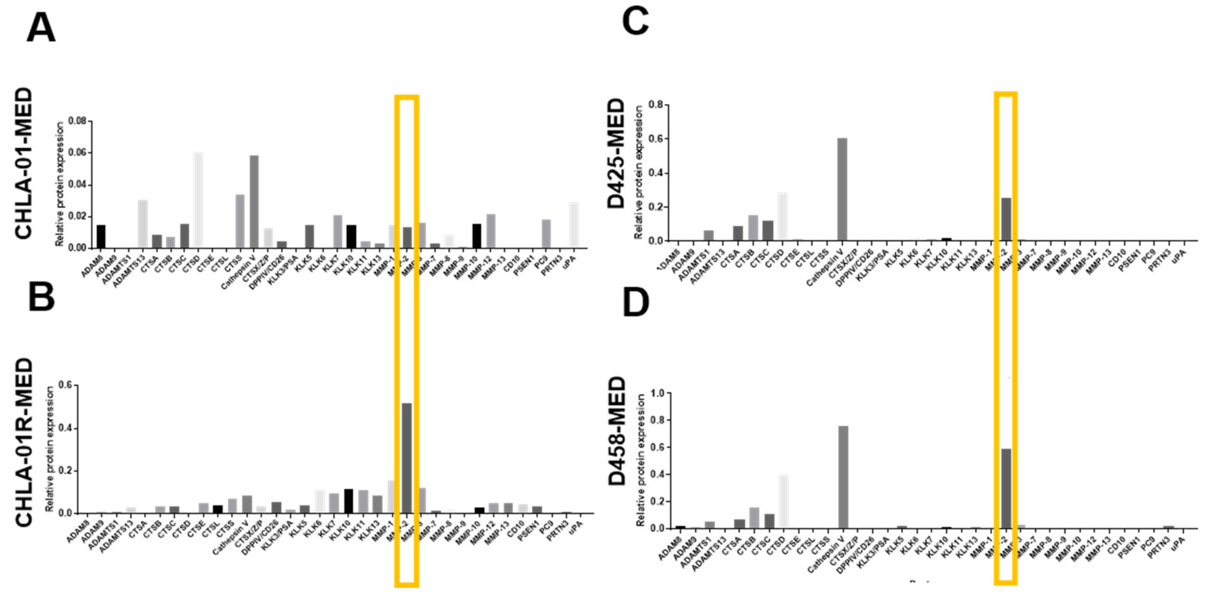

Supplementary Figure S3. Densitometry of protease microarrays of the matched Group 3/4 medulloblastoma cell lines. (A) The Group 4 primary (CHLA-01-Med) and (B) the group 4 metastatic (CHLA-01R-Med) cell lines express different levels of MMP-2 (yellow box; CHLA-01-Med =0.014, CHLA-01R-Med=0.516, a 37-fold increase). (C) The group 3 primary (D425-Med) and (D) the group 3 metastatic (D458-Med) cell lines also express different levels of MMP-2 (yellow box D425-Med = 0.255 D458-Med=0.592, a 2-fold increase). Signal intensities were quantified using a microarray plugin from Image J software, demonstrating differential expression of analytes relative to the signal intensities of reference dots on each membrane.

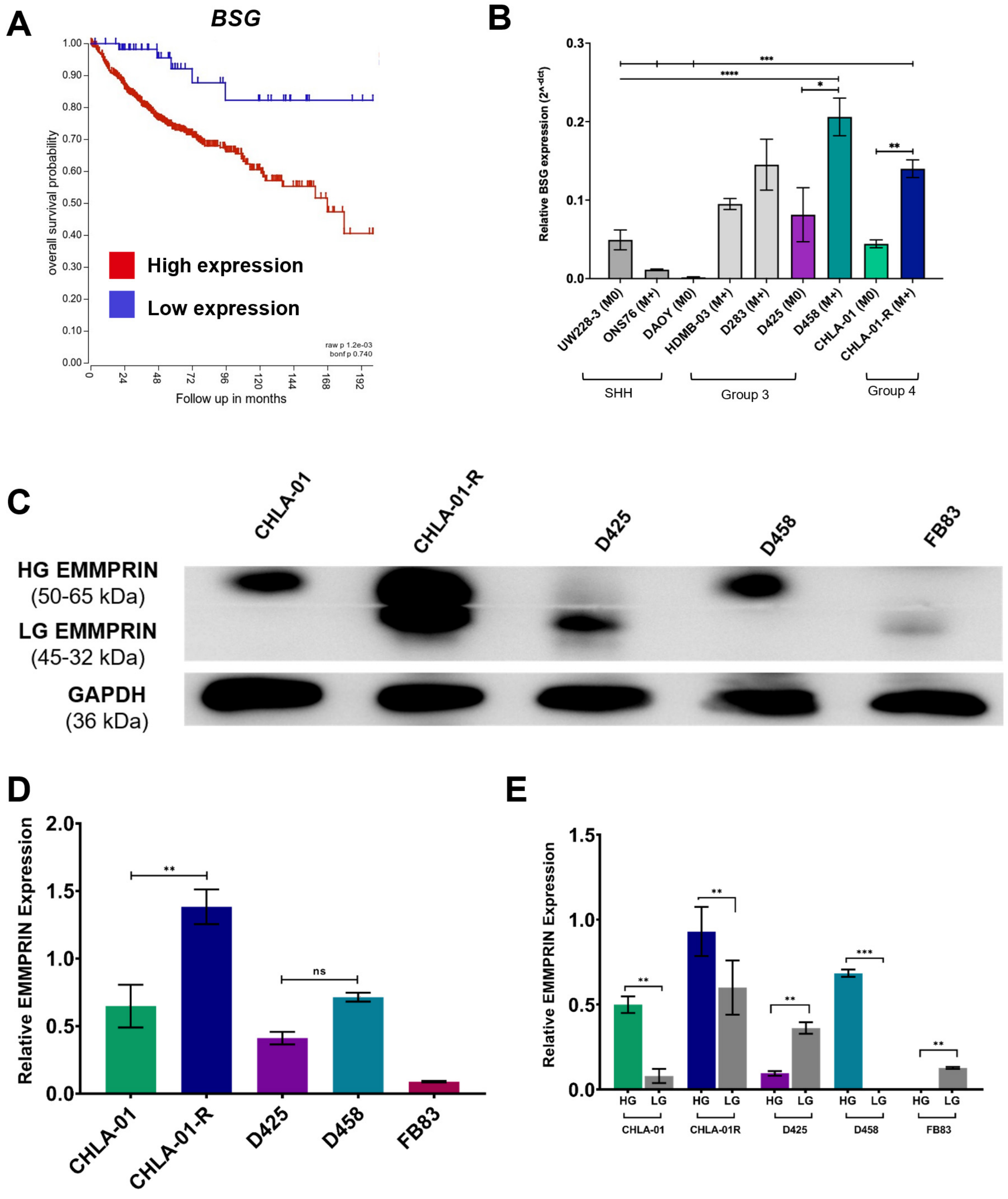

Supplementary Figure S4. EMMPRIN gene and protein expression in medulloblastoma. (A) Kaplan-Meier survival analysis of *BSG* (the gene encoding EMMPRIN) in the Cavalli dataset of 766 medulloblastoma patients shows a clear prognostic significance of high expression correlating with poor overall survival. Survival curves were compared using the Log-rank (Mantel-Cox) test. (**B)** *BSG* expression was determined in a panel of medulloblastoma cell lines by qPCR. Expression was significantly higher in metastatic cell lines compared to matched primary cell lines. EMMPRIN protein expression was assessed in metastatic and primary cell lines and a non-cancerous control line (FB83) by western blotting SHH (sonic hedgehog). A representative western blot is shown in (**C**); band intensity analysis of independent western blots allowed quantitation of total (**D**) and differently glycosylated forms of EMMPRIN (**E**), showing significantly higher levels of high molecular weight EMMPRIN in metastatic cell lines. Significance was calculated using one-way ANOVA analyses with Dunnett's multiple comparisons post-hoc test, (**p≤0.05, **p≤0.01, ***p≤0.005, ***p≤0.001) (ns=not significant). Data represent the

average of three independent experiments with error bars indicating standard error of the mean (SEM). The uncropped blots are shown in File S1.

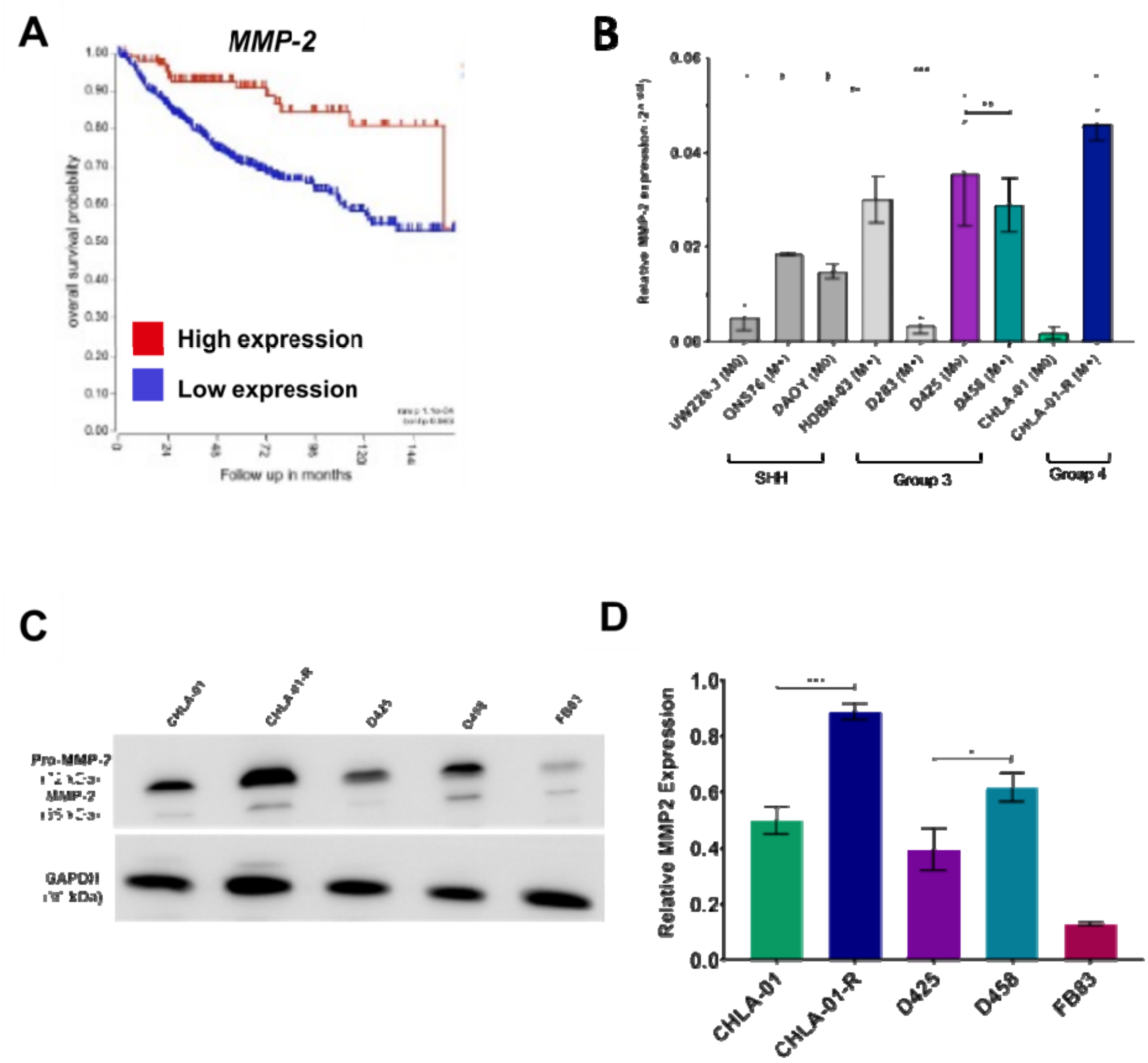

Supplementary Figure S5. MMP-2 gene and protein expression in medulloblastoma. (A) Kaplan-Meier survival analysis of MMP-2 in the Cavalli dataset of 766 medulloblastoma patients shows low expression correlating with poor overall survival. Survival curves were compared using the Log-rank (Mantel-Cox) test. (**B)** *MMP-2* expression was determined in a panel of medulloblastoma cell lines by qPCR and showed no consistent pattern of differential expression either with respect to medulloblastoma subgroup or between paired cell lines SHH (sonic hedgehog). MMP-2 protein expression was assessed in metastatic and primary cell lines and a non-cancerous control line (FB83) by western blotting. A representative western blot is shown in (**C**); band intensity analysis of independent western blots allowed quantitation of MMP-2 protein expression (**D**) showing significantly higher levels of MMP-2 in metastatic cell lines compared to their matched primary cell line. Significance was calculated using one-way ANOVA analyses with Dunnett's multiple comparisons post-hoc test, (**p≤0.05, **p≤0.01, ***p≤0.005, ***p≤0.001) (ns=not significant). Data represent the average of three independent experiments with error bars indicating standard error of the mean (SEM). The uncropped blots are shown in File S1.

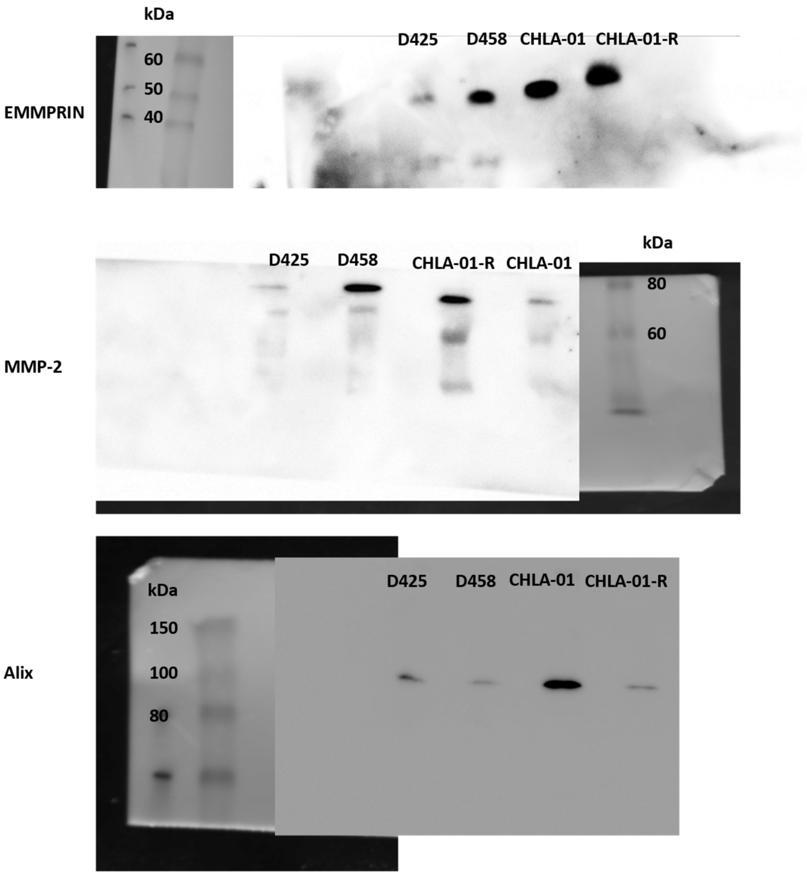

Supplementary Figure S6. Representative full-length western blot images from Figure 3A confirming exosomal EMMPRIN and MMP2 protein expression. Alix was used as a loading control.

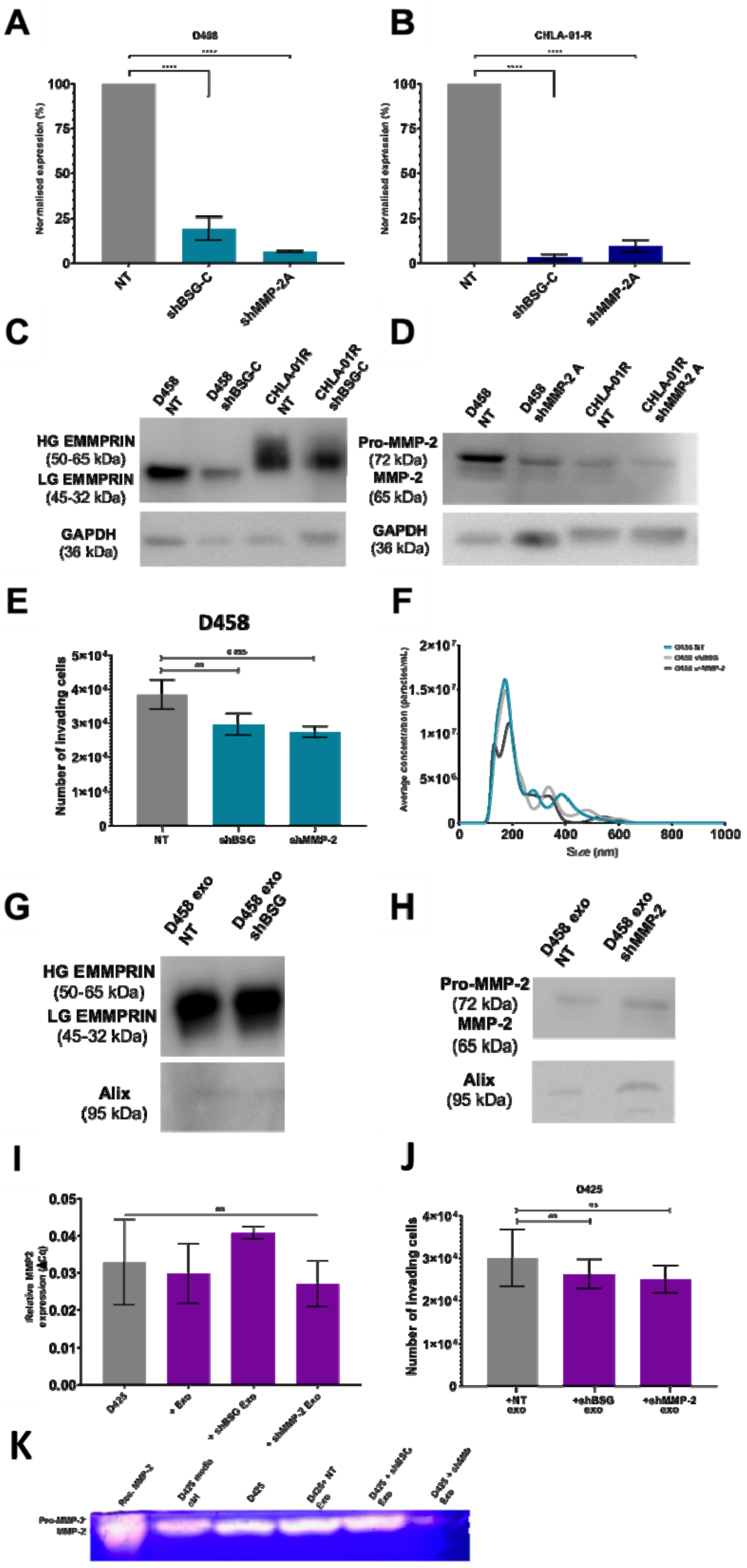
A
D458
Normalised expression (%)
NT
shBSG-C
shMMP-2A
B
CHLA-01-R
Normalised expression (%)
NT
shBSG-C
shMMP-2A
C
D458 NT
D458 shBSG-C
CHLA01R NT
CHLA01R shBSGC
HG EMMPRIN (50-65 kDa)
LG EMMPRIN (45-32 kDa)
GAPDH (36 kDa)
D
D458 NT
D458 shMMP-2 A
CHLA-01R NT
CHLA-01R shMMP-2 A
Pro-MMP-2 (72 kDa)
MMP-2 (65 kDa)
GAPDH (36 kDa)
E
D458
Number of invading cells
NT
shBSG
shMMP-2
F
Average concentration (particles/mL)
Size (nm)
G
D458 exo NT
D458 exo shBSG
HG EMMPRIN (50-65 kDa)
LG EMMPRIN (45-32 kDa)
Alix (95 kDa)
H
D458 exo NT
D458 exo shMMP-2
Pro-MMP-2 (72 kDa)
MMP-2 (65 kDa)
Alix (95 kDa)
I
Relative MMP2 expression (ΔCt)
D425
+ Exo
+ shBSG Exo
+ shMMP-2 Exo
J
D425
Number of invading cells
+NT exo
+shBSG exo
+shMMP-2 exo
K
Pro-MMP-2
MMP-2

Supplementary Figure S7. ***BSG* and *MMP-2* were significantly depleted using shRNA in D458 and CHLA-01-R cell lines.** Relative *BSG* (A) and *MMP-2* (B) mRNA expression displayed as fold change (ΔCq) relative to the appropriate non-silencing control. Western blot analysis revealed corresponding protein expression to be depleted in the knockdown cell lines D458 (C) and CHLA-01R (D). Group 3 cell invasion through a collagen and laminin IV coated transwell chamber insert was quantified by PrestoBlue metabolic staining and compared to cell lines transduced with non-targeting (NT) (E). Exosomes from D458 knockdown cell lines and non-transduced cells were isolated and their size and concentration was measured by NanoFCM (F). Western blot analysis revealed both EMMPRIN (G) and MMP-2 (H) protein levels to be depleted in exosomes isolated from knockdown cell lines. Exosomes from D458 knockdown cell lines were applied to the matched parental cell line D425 (+NT exo: non-targeting, +shBSG exo, knockdown BSG or shMMP-2 exo knockdown MMP-2) following which *MMP-2* mRNA expression levels in recipient cells was determined by qRT-PCR (I). The ability of these recipient cell lines to invade through a laminin and collage IV matrix was determined by metabolic assay (J). MMP-2 activity in the medium of D425 cells receiving exosomes was determined by gelatin zymography. Proteolysis was detected as a white band (K). Significance was assessed by ordinary one-way ANOVA analysis with Sidak's multiple comparison tests (**p≤0.05, **p≤0.01, ***p≤0.005, ****p≤0.001) (ns=not significant). Data represent the average of at least two independent experiments with error bars indicating standard error of the mean (SEM). The uncropped blots are shown in File S1.
