# Supplementary material for: Extracellular Vesicles Potentiate Medulloblastoma Metastasis in an EMMPRIN and MMP-2 Dependent Manner
Source: Cancers (Basel). 2023 May 4;15(9):2601. doi: 10.3390/cancers15092601 (PMC10177484; doi:10.3390/cancers15092601)
Supplement: Supplementary file 1 [file cancers-15-02601-s001.zip › File S1. Full length western blots- resubmission final.pptx]

## Slide 1
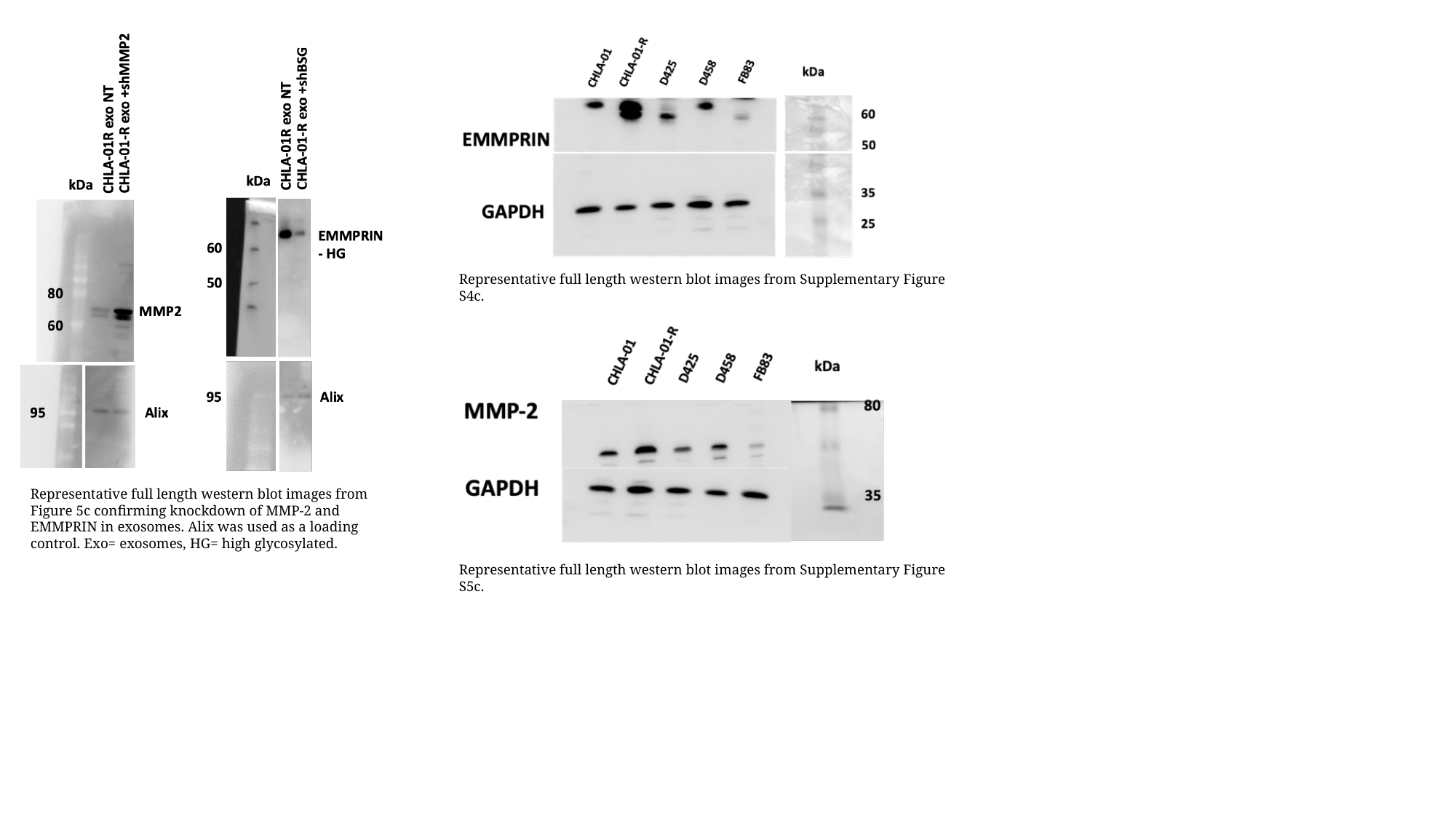

Representative full length western blot images from Supplementary Figure S4c.
Representative full length western blot images from Figure 5c confirming knockdown of MMP-2 and EMMPRIN in exosomes. Alix was used as a loading control. Exo= exosomes, HG= high glycosylated.
Representative full length western blot images from Supplementary Figure S5c.

## Slide 2
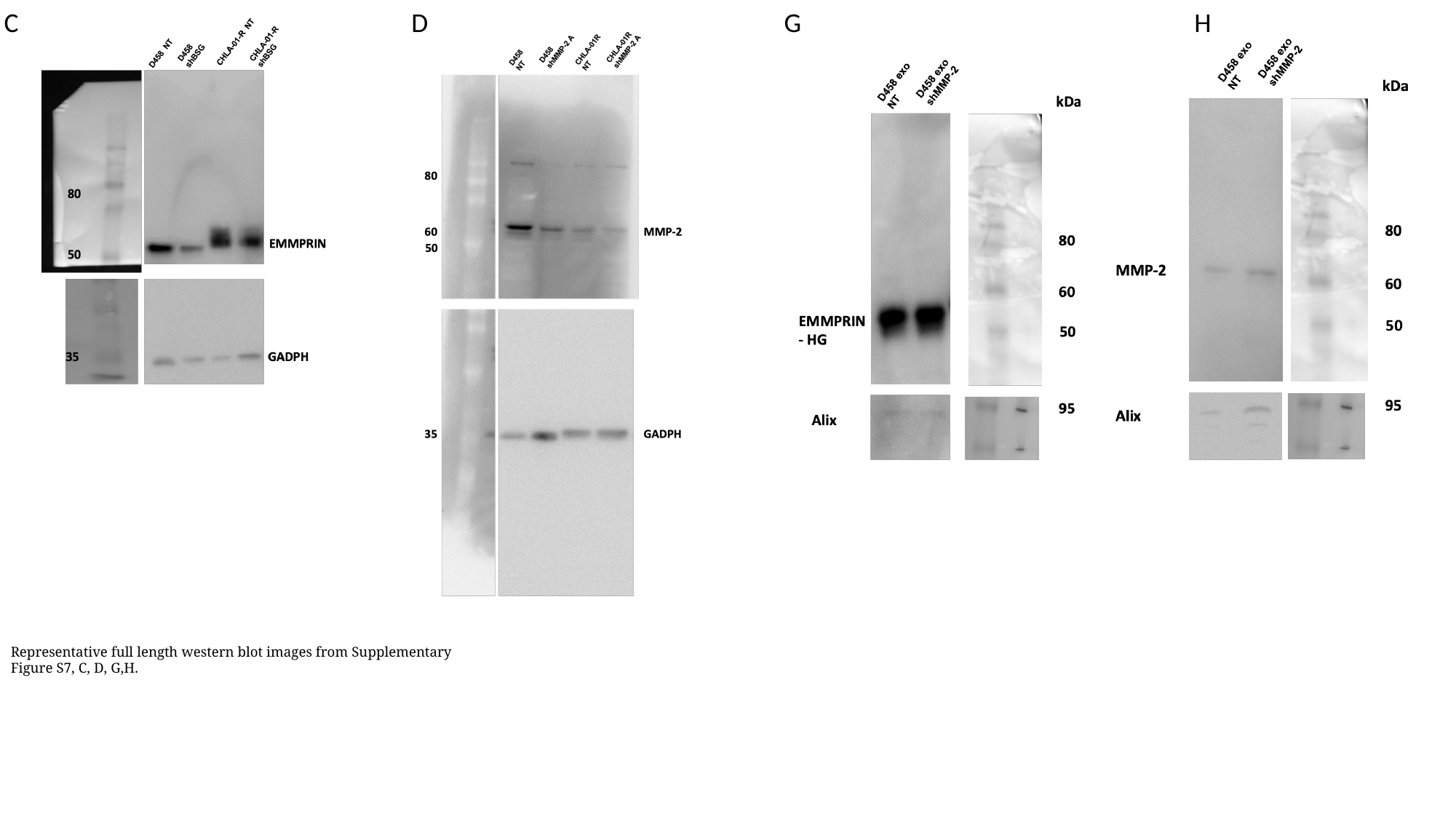

C
D
G
H
Representative full length western blot images from Supplementary Figure S7, C, D, G,H.

## Slide 3
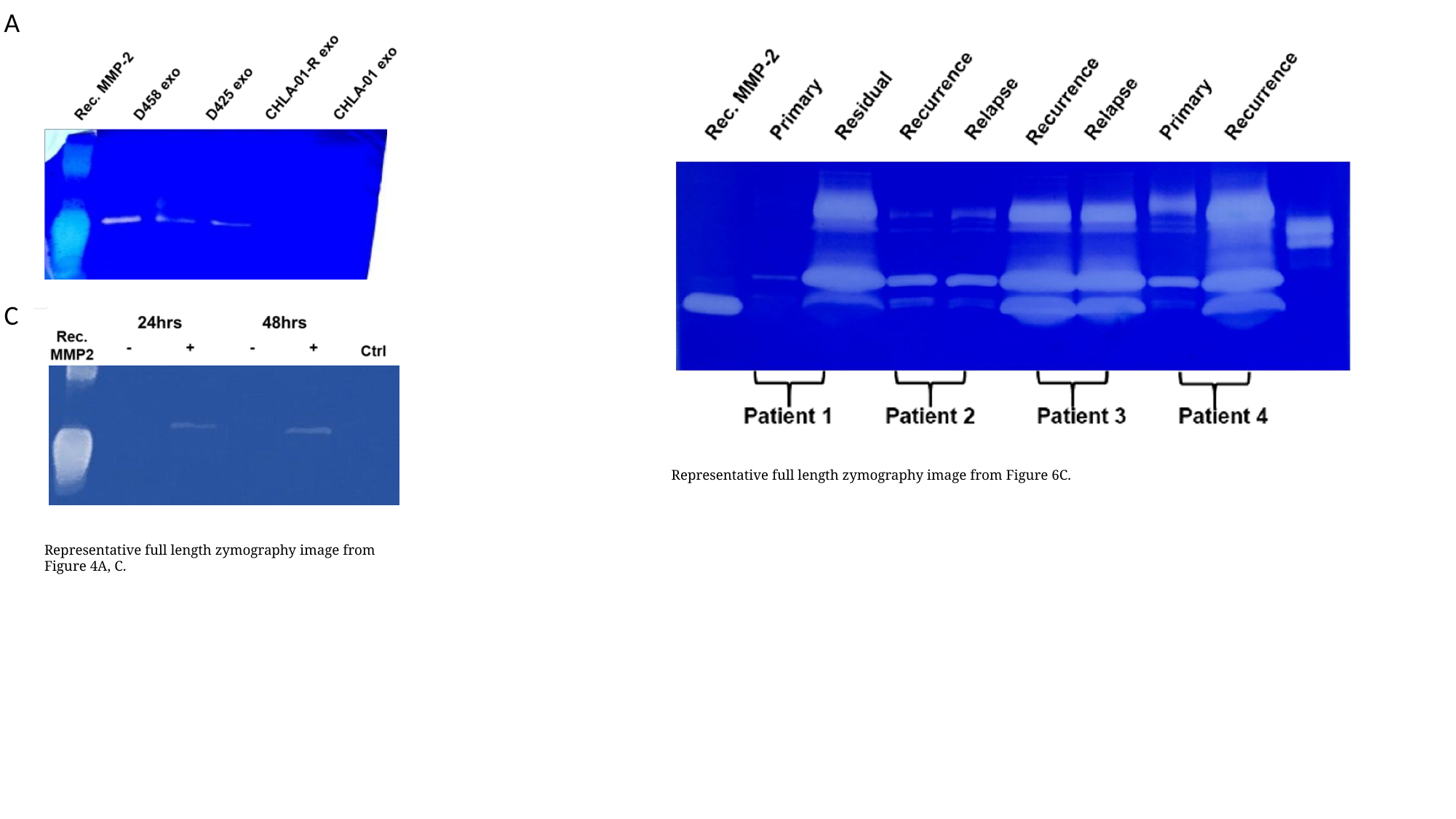

A
C
Representative full length zymography image from Figure 6C.
Representative full length zymography image from Figure 4A, C.

## Slide 4
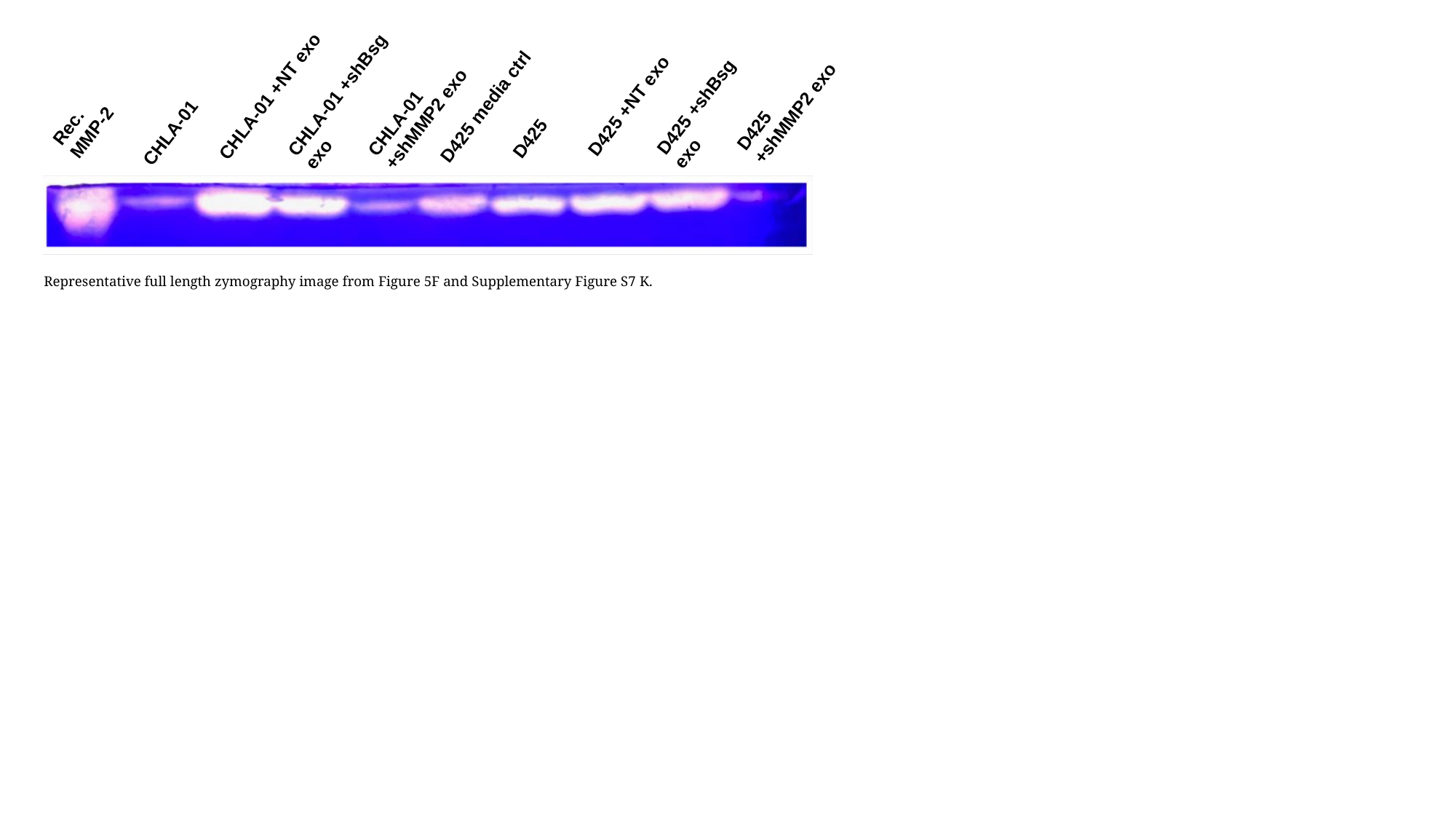

D425
CHLA-01 +NT exo
CHLA-01 +shBsg exo
D425 media ctrl
D425 +shMMP2 exo
CHLA-01
D425 +shBsg exo
CHLA-01 +shMMP2 exo
Rec. MMP-2
D425 +NT exo
Representative full length zymography image from Figure 5F and Supplementary Figure S7 K.
