# Supplementary material for: Extracellular Vesicles Potentiate Medulloblastoma Metastasis in an EMMPRIN and MMP-2 Dependent Manner
Source: Cancers (Basel). 2023 May 4;15(9):2601. doi: 10.3390/cancers15092601 (PMC10177484; doi:10.3390/cancers15092601)
Supplement: Supplementary file 1 [file cancers-15-02601-s001.zip › Jackson et al. Supplementary methods.pdf]

Supplementary methods

Protease arrays

Forty-eight hour conditioned cell supernatants were tested immediately after being harvested, using a Human Protease Array kit (R&D Systems; ARY025). Reagents and buffers required were all provided within the kit and used according to the manufacturer's recommendations. Briefly, nitrocellulose membranes were placed into a multi-well plastic dish and incubated in the provided blocking buffer for an hour on a shaker at room temperature. During this time, samples for analysis were diluted to a volume of 1.5ml with array buffer 6, with the addition of 15µl of the protease detection antibody cocktail. The membranes were then incubated in the supernatant/array buffer 6 solution overnight at 4˚C. The next day, in a manner similar to western blotting, the membranes were thrice-washed before being incubated with 2mL of Streptavidin-HRP. Further washing with the washing buffer was done after 30 minutes of incubation and signal detected using chemiluminescence. Array correlates were localised using the transparent overlay templates provided and quantified using a publicly available automatic macro for analysis of protein arrays ('Protein Analyzer', https://imagej.nih.gov/ij/macros/toolsets/Protein Array Analyzer.txt). The protein levels were determined by converting pixel densities to relative light units using ImageJ and thereby quantifying the protein signals for each condition. Densitometry performed on Image J was then used to compare the relative protease expression within the supernatant samples, with normalisation to the mean of the reference controls on each membrane. Graphs were created using GraphPad Prism 8 (GraphPad Software Inc., La Jolla, CA, USA).
